# COVID-19 and financial toxicity in patients with renal cell carcinoma

**DOI:** 10.1007/s00345-020-03476-6

**Published:** 2020-10-22

**Authors:** Michael D. Staehler, Dena J. Battle, Cristiane D. Bergerot, Sumanta Kumar Pal, David F. Penson

**Affiliations:** 1 Department of Urology, Ludwig-Maximilians University, University of Munich, Marchioninistr. 15, 81377 Munich, Germany; 2 Kidney Cancer Research Alliance (KCCure), Alexandria, VA USA; 3 Department of Medical Oncology and Experimental Therapeutics, City of Hope Comprehensive Cancer Center, Duarte, CA USA; 4 Department of Urology, Vanderbilt University Medical Center, Nashville, TN USA

**Keywords:** Renal cell carcinoma, Health care survey, Frustration, Qualitative study, Financial toxicity, COST score

## Abstract

**Purpose:**

To ascertain renal cell carcinoma (RCC) financial toxicity on COVID-19 during the COVID-19 crisis as patients are struggling with therapeutic and financial implications.

**Methods:**

An online survey was conducted from March 22 to March 25, 2020. It included baseline demographic, clinicopathologic, treatment-related information, anxiety levels related to COVID-19, questions related to financial concerns about COVID-19 as well as the validated 11-item COST measure.

**Results:**

Five-hundred-and-thirty-nine patients (39%:58% male:female) from 14 countries responded. 23% of the patients did not feel in control of their financial situation but 8% reported being very satisfied with their finances. The median COST score was 21.5 (range 1–44). Metastatic patients who have not started systemic therapy had a COST score (19.8 range 2–41) versus patients on oral systemic therapy had a COST score (23.9 range 4–44). Patients in follow-up after surgery had a median COST score at 20.8 (range 1–40). A low COST scores correlated (*p* < 0.001) were female gender (*r* = 0.108), younger age (*r* = 0.210), urban living situation (*r* = 0.68), a lower educational level (*r* = 0.155), lower income (*r* = 0.165), higher anxiety about acquiring COVID-19 (*r* = 0.198), having metastatic disease (*r* = 0.073) and a higher distress score about cancer progression (*r* = 0.224).

**Conclusion:**

Our data highlight severe financial impact of COVID-19. Acknowledging financial hardship and thorough counseling of cancer patients should be part of the conversation during the pandemic. Treatment and surveillance of RCC patients might have to be adjusted to contemplate financial and medical needs.

**Electronic supplementary material:**

The online version of this article (10.1007/s00345-020-03476-6) contains supplementary material, which is available to authorized users.

## Introduction

Much has been written about how the COVID-19 crisis is placing immense strain on healthcare systems worldwide. As a consequence, there has been a rush to develop guidelines around every element of cancer care, ranging from use of surgery, radiation, systemic therapy to supportive care modalities [1–4]. Practice changes have been implemented to reduce touchpoints for patients and to preserve hospital resources for COVID-19 patients. However, less research has focused on how the crisis is impacting individuals being treated for cancer, including adverse effects of financial toxicity.

Advances in care for patients with renal cell carcinoma have dramatically changed over the last decade, improving outcomes and increasing overall survival for patients. However, these advances have occurred during a time when insurance plans have also increased cost-sharing, shifting a greater proportion of treatment costs directly to the patient [5].

An estimated 2.7 billion people have been impacted by coronavirus lockdowns and according to the United Nations Conference on Trade and Development, the global economy could lose $2 trillion as a result of the crisis [6].

Advances in cancer care become meaningless if patients cannot afford them or if the patients’ prognoses continue to be determined by where they live or how good their insurance is [7].

We conducted an online survey to ascertain (a) patient anxiety level around COVID-19 and (b) implications on financial burden.

## Materials and methods

### Survey development and distribution

The survey was developed by the Kidney Cancer Research Alliance (KCCure), with multidisciplinary representation from two surgeons (MS, DP), medical oncologist (SKP), psychologist (CB) and patient advocate (DB). The survey included a total of 45 items (detailed subsequently) and was initially evaluated by a separate group of patient advocates for ease of interpretability. The open survey was then broadcast to the KCCure membership through a patient mailing list of *n* = 1532 subscribers maintained by the organization and was also distributed through online social media platforms (specifically, Facebook and Twitter). Multiple responses from the same patient were prohibited by the system via anonymized IP address tracking. IRB was not obtained as the survey was a patient organization’s effort. No personal data were collected in the de-personalized questionnaire and prior to taking the survey patients gave informed consent for data use and analysis.

### Survey composition

The survey is included as Supplementary Appendix 1. Briefly, the survey included demographic features including age, gender, race, educational level and income level. Patients were queried regarding their perceived risk of COVID-19, and their anxiety level related to both COVID-19 and cancer progression was quantified using a Likert scale ranging from 0 to 10.

Further questions were based on disease status. Patients in surveillance were queried regarding their current plan for surveillance. Patients receiving systemic treatment were queried regarding the nature of systemic therapy they were receiving.

The survey contained items pertaining to distress level, financial hardship, medical and behavioral expectations that were quantified on a Likert scale ranging from 0 to 3. To assess financial burden, the validated comprehensive score for financial toxicity (COST) patient-reported outcome measure was included. The lower the score, the worse the financial toxicity [8].

### Statistical analysis

Descriptive statistics with graphical outputs were used to characterize survey results.

Pearson’s correlation (*r*) and Kendal’s tau test were used to analyze the COST questionnaire, financial burden and hardship, as well as medical and behavioral expectations. Significance levels were two-tailed. All statistical analyses were carried out with SPSS Statistics Vers. 26 (IBM Analytics, Armonk, NY, USA).

## Results

### Patient characteristics

Responses were received via e-mail and social media. With a response rate of 35% and 539 total respondents, 280 patients (52%) had metastatic disease, 187 patients (35%) had prior surgery for localized disease and 23 patients (5%) had localized disease awaiting surgery (Table 1). Median age was 55 (range, 24–87) with 58% females and 39% males. Most patients had obtained a bachelor or graduate degree (44%) and live in the United States (87%). In addition, the majority of patients are receiving treatment at an academic center (37%), followed by regional centers (30%) and private practices (18%). Patients were predominantly white (88%) and well educated (58% had college or graduate degree. The majority had a household income that ranged between $50,000 and $99,999 (30%) or higher than $100,000 (38%). The majority of participants live in suburban areas (52%). Socioeconomic and clinical characteristics were statistically not significantly different between groups and are described in Table 1.

**Table 1:** Patient characteristics

	Localized (no surgery), *n* = 23	Localized (prior surgery), *n* = 187	Metastatic, *n* = 280	Total, *n* = 539
Age	46 (24–67)	52 (26–86)	57 (31–87)	55 (24–87)
Gender				
Male	6 (26.1%)	49 (26.2%)	134 (47.8%)	310 (57.5%)
Female	14 (60.8%)	134 (71.6%)	137 (48.9%)	211 (39.1%)
Race				
White	19 (82.6%)	165 (88.2%)	247 (88.2%)	473 (87.8%)
Hispanic or Latino	0 (0.0%)	7 (3.7%)	11 (3.9%)	20 (3.7%)
Black/African American	0 (0.0%)	4 (2.1%)	3 (1.1%)	7 (1.3%)
Asian/Pacific Islander	0 (0.0%)	6 (3.2%)	7 (2.5%)	17 (3.2%)
Native American	0 (0.0%)	2 (1.1%)	1 (0.3%)	3 (0.6%)
Other	0 (0.0%)	3 (1.6%)	5 (1.8%)	10 (1.9%)
Type of practice				
Academic center	7 (30.4%)	57 (30.4%)	110 (39.3%)	199 (36.9%)
Regional center	7 (30.4%)	47 (25.1%)	98 (35.0%)	162 (30.1%)
Community hospital	3 (13.0%)	29 (15.5%)	30 (10.7%)	69 (12.8%)
Private practice	3 (13.0%)	53 (28.3%)	38 (13.6%)	98 (18.2%)
Education level				
Less than high school	0 (0%)	3 (1.6%)	5 (1.8%)	9 (1.7%)
High school	3 (13.0%)	26 (13.9%)	41 (14.6%)	81 (15.0%)
Some college	6 (26.1%)	50 (26.7%)	65 (23.2%)	128 (23.7%)
College/graduate degree	10 (43.5%)	107 (57.2%)	164 (58.6%)	310 (57.5%)
Income level				
$0–$24,999	3 (13.0%)	18 (9.6%)	20 (7.1%)	46 (8.5%)
$25,000–$49,999	4 (17.4%)	35 (1.6%)	39 (13.9%)	85 (15.8%)
$50,000–$99,999	6 (26.1%)	61 (32.6%)	85 (30.4%)	164 (30.4%)
$100,000+	7 (30.4%)	60 (32.1%)	117 (41.8%)	203 (37.7%)

### COVID-19 and expected hardships

The majority of patients expect the pandemic to induce medical, behavioral, psychological and financial hardship. Only 17% do not expect medical, 25% behavioral, 16% psychological and 13% financial hardship to occur as a result of COVID-19. Patients with an income of less than US $50,000 were significantly more anxious about financial hardship than people with a high income (> US $100,000) (93% vs 82%, *p* < 0.001).

Patients with a higher anxiety about acquiring COVID-19 were significantly (*p* < 0.001) more likely to expect medical (*r* = 0.212), behavioral (*r* = 0.178) and psychological hardship (*r* = 0.304).

### Financial anxiety

The majority of patients (59%) were concerned that their money in savings or retirements assets would not cover the cost of their treatment. Only 8% were satisfied with their financial situation and 25% were financially stressed. More than a quarter of the patients (26%) were concerned about losing their job and income and 32% claim that their cancer treatment has reduced their satisfaction with their present financial situation. Half of the patients do not feel in control of their financial situation and only 8% were confident about their finances.

### Educational and income level

Patients with a higher educational level or a higher income were significantly more often living in urban areas and were more likely to be treated at an academic center (*p* < 0.001). Compared to patients with a lower educational or income level, they did not have higher anxiety related to acquiring COVID-19 and had the same level of cancer distress.

### COST score assessment

**Fig. 1:**
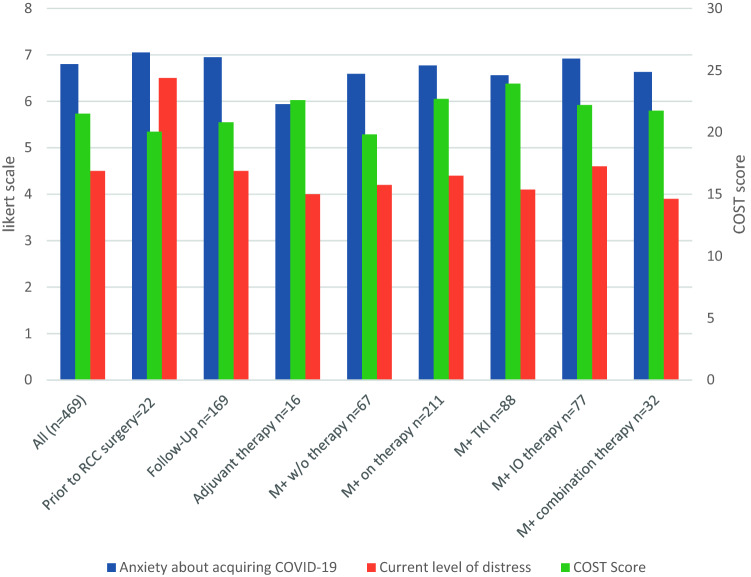
COST score and anxiety related to different situations

The median COST score of all patients was 21.5 (range 1–44). Detailed responses are shown in Table 2. Metastatic patients who have not started systemic therapy had the lowest median COST score (19.8 range 2–41) versus patients on systemic therapy with oral treatments had the highest median COST score (23.9 range 4–44). Interestingly, patients in follow-up after surgery also had a lower median COST score at 20.8 (range 1–40) (see Fig. 1).

**Table 2:** COST responses – all patients

	Not at all % *N*	A little bit % *N*	Somewhat % *N*	Quite a bit % *N*	Very much % *N*
I know that I have enough money in savings retirements or assets to cover the cost of my treatment	42% 200	17% 81	23% 109	9% 44	9% 45
My out-of-pocket medical expenses are more than I thought they would be	28% 135	22% 103	24% 114	14% 69	12% 58
I worry about the financial problems I will have in the future as a result of my illness or treatment	14% 68	20% 95	24% 117	21% 100	21% 100
I feel I have no choice about the amount of money I spend on care	12% 59	11% 51	27% 127	22% 106	28% 136
I am frustrated that I cannot work or contribute as much as I usually do	30% 142	16% 78	16% 75	17.09% 81	20.68% 98
I am satisfied with my current financial situation	27% 128	18% 88	36% 171	13% 62	6% 31
I am able to meet my monthly expenses	8% 37	12% 59	32% 152	24% 114	25% 118
I feel financially stressed	15% 73	29% 138	30% 142	13% 60	14% 66
I am concerned about keeping my job and income, including work at home	39% 179	14% 64	20% 90	12% 56	15% 71
My cancer or treatment has reduced my satisfaction with my present financial situation	23% 110	24% 112	21% 100	16% 77	16% 75
I feel in control of my financial situation	23% 111	22% 107	34% 163	12% 58	9% 41

**Fig. 2:**
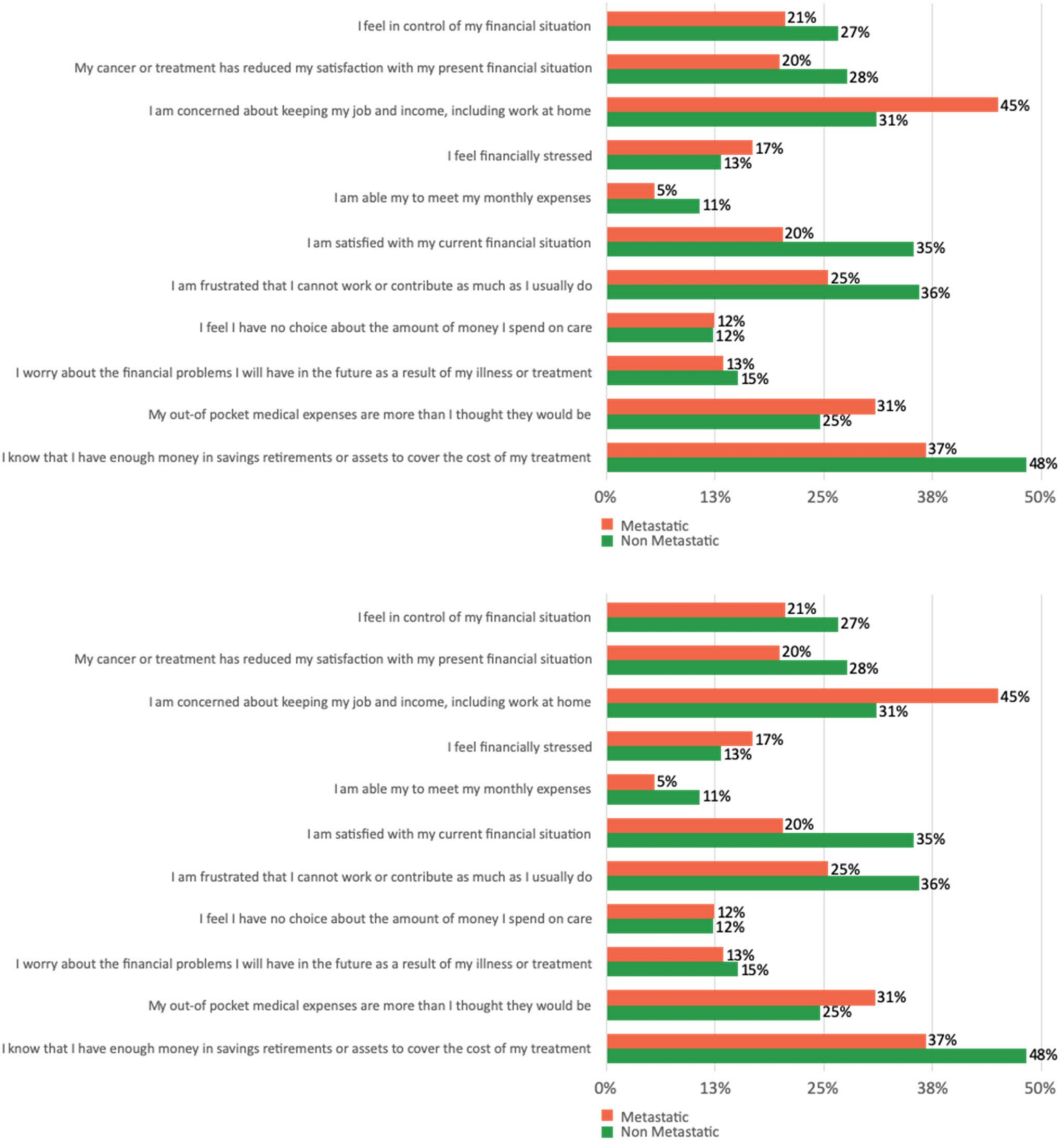
COST Score with selection “not at all” comparing different topics between metastatic and non-metastatic respondents

Factors significantly (*p* < 0.001) contributing to a lower COST score were female gender (*r* = 0.108), younger age (*r* = 0.210), urban living situation (*r* = 0.68), a lower educational level (*r* = 0.155), lower income (*r* = 0.165), higher anxiety about acquiring COVID-19 (*r* = 0.198), having metastatic disease (*r* = 0.073) and a higher distress score about cancer progression (*r* = 0.224) (see Fig. 2).

Nationality and race were not correlated with financial distress in our dataset.

## Discussion

Our study reveals that while patients experience heightened anxiety about increasing financial hardship related to COVID-19, they remain substantially concerned about cancer recurrence and progression. In the overall population, 71% felt they had a heightened risk for COVID-19 infection; however, only 27% of patients contacted their treating physicians to confirm this information. Patients with RCC seem unwilling to compromise planned surveillance for localized disease or planned systemic therapy for metastatic disease.

In our cohort, anxiety related to financial hardship is very high, mainly in patients with a moderate-to-high income and higher educational level.

The median COST score in our cohort of RCC patients is 21.5, which is somewhat lower than previously described cohorts of cancer patients in general, where it was at 23, indicating that pandemic might be increasing financial anxiety [8–10].

Although oral systemic therapy based on tyrosine-kinase inhibitors has one of the highest co-pays for patients [9], this was the subgroup of respondents that expected the lowest financial hardship with a COST score of 23.9. Patients prior to surgery had the one of the lowest scores at 20.0, which could be a reflection of the fear of future costs versus actual cost. The lower rates of financial concern for patients receiving oral therapy could be due to the fact that these drugs are more commonly used in later-line treatment, reflecting a patient population that has more experience with treatment resulting in lower anxiety levels related to cost. In line with previous findings, younger patients were more likely to be anxious about financial hardship and might need additional counseling and a specific plan to address this anxiety [9].

Interestingly, patients who have been diagnosed with cancer, but have not yet had treatment, expressed higher anxiety about financial toxicity versus patients already on therapy. This was true for both metastatic patients and patients with localized disease. It could be that uncertainty related to future costs is a larger driver of financial toxicity rather than the actual realized cost of treatments for patients. This might indicate a need for increased counseling for patients who are newly diagnosed and could be experiencing anxiety due to the uncertainty related to potential financial costs (Fig. 2).

This concern related to uncertainty of future costs is also evident in the responses related to the COST questionnaire. When asked about confidence in covering the cost of treatment, 41% indicate that they are not at all confident that they have enough money in their savings or retirement. Yet, when asked whether their out-of-pocket expenses were more than they thought they would be, only 12% said “very much”. Only 6% of patients said that they feel satisfied with their current situation, but when asked if they were able to meet their monthly expenses, only 8% indicated that they were not able to do so. When asked if they felt in control of their financial situation, less than 10% indicated that they were. But again, when asked if their cancer or treatment had reduced their satisfaction with their present financial situation, only 16% said very much.

Uncertainty in cancer is one of the most significant forms of distress in cancer patients [11]. It could be that the specter of future costs, fear related to inability to care for their family as their disease progresses, could be more important drivers to consider when measuring financial toxicity.

Research related to patient-reported outcomes, while rapidly advancing in cancer care, is still poorly understood in renal cell carcinoma. Despite demands from patient advocacy organizations, patient values concerning treatment decisions are yet to be defined and poorly described and understood [12]. Repeated assessment and analyses to differentiate trends from principles are needed. Nonetheless, the high rates of anxiety highlight the need to address financial toxicity as a standard part of care for kidney cancer patients.

Comparing these numbers needs to be done with caution as there has not been a COST analysis in RCC so far. As the COST score observed in our patient cohort is comparable to other disease areas, we are confident it reflects on the real situation. As there is uncertainty and the fear of losing jobs and access to medical care is settling in, we believe it reflects the actual changes in the medical situation rather than being caused by a selection bias. Notably, reported cohorts so far had comparable demographics and baseline characteristics, with the majority of respondents being female, having a higher educational level and income [9, 10].

Limitations of our study include the use of data supplied by patients. Using this approach, confirmation of medical data (e.g., histology, stage, treatment regimen) is not feasible. Furthermore, the questionnaire outside the COST data was not validated. There is also likely some selection bias among survey respondents—our population was predominantly female, highly educated and primarily US-based, and a relatively high proportion was treated at academic centers. But compared to previous surveys, in our patient cohort with more than 1500 responses, no differences in characteristics were seen (data submitted for publication). Perhaps most importantly, the data for COVID-19 are evolving extremely rapidly. The distribution of cases is changing, as is the approach to infection prevention, prophylaxis and treatment [1, 3, 13–16]. As such, it is possible that the perspective of patients and physicians will change as the situation progresses. To supplement this, we plan to continue to survey the RCC community to obtain the patient perspective on management.

## Conclusion

Our data highlight severe financial impact of COVID-19 in patients with RCC. Acknowledging financial hardship and uncertainty related to future costs and providing thorough counseling of cancer patients should be part of the conversation during the pandemic. Younger patients, women and those with a lower income seem to be at highest need of financial advising. Treatment and surveillance of RCC patients might have to be adjusted to contemplate financial and medical needs.

## Electronic supplementary material

Below is the link to the electronic supplementary material.

Supplementary file1 (PDF 372 kb)
