# Supplementary material for: COVID-19 and financial toxicity in patients with renal cell carcinoma
Source: World J Urol. 2020 Oct 22;39(7):2559–65. doi: 10.1007/s00345-020-03476-6 (PMC7578440; doi:10.1007/s00345-020-03476-6)
Supplement: Supplementary file 1 — Supplementary file1 (PDF 372 kb) [file 345_2020_3476_MOESM1_ESM.pdf]

Introduction

The recent COVID-19 crisis is causing significant issues and concerns for kidney cancer patients. This survey is intended to understand patient experiences related to the outbreak and how it is impacting care.

KCCure surveys are designed to ensure that the patient voice is effectively communicated to providers, industry leaders and policy makers. All answers are used anonymously and analyzed according to scientific standards by KCCure in conjunction with medical scientists. None of your personal data will be revealed or used other than to calculate statistics.

If you are taking this survey as a caregiver on behalf of a patient, please respond as if you were the patient (personal data, age etc.).

Thank you for your support

The KCCure Team

About you

****** if you are filling out the survey on behalf of a patient please respond to all questions as if you were the patient ******

1. Please choose:

- ◯ I am a patient
- ◯ I am a taking this survey on behalf of a patient

2. If you are a caregiver, select who you are caring:

- ◯ Spouse
- ◯ Child
- ◯ Parent
- ◯ Sibbling
- ◯ Other (please specify)

3. What is your sex? (if you are filling out the survey on behalf of a patient please respond as if you were the patient)

- ◯ Female
- ◯ Male

4. Tell us your age (enter birth date below)

Date / Time

Date

MM/DD/YYYY

5. Are you White, Hispanic or Latino, Black or African-American, Asian/Pacific Islander, American Indian or Native American, or some other race?

- White
- Hispanic or Latino
- Black or African-American
- Asian/Pacific Islander
- American Indian or Native American
- Other or prefer not to disclose

6. Which best describes where you live

- Urban
- Suburban
- Rural

7. What country do you live in?

8. If you selected the United States, what state or U.S. territory do you live in?

9. What is the highest level of school you have completed or the highest degree you have received?

- Less than high school degree
- High school degree or equivalent (e.g., GED)
- Some college but no degree
- Associate degree
- Bachelor degree
- Graduate degree

10. What is your approximate average household income?

- $0-$24,999
- $25,000-$49,999
- $50,000-$74,999
- $75,000-$99,999
- $100,000-$124,999
- $125,000-$149,999
- $150,000-$174,999
- $175,000-$199,999
- $200,000 and up

11. Which answer best describes the center where you are currently receiving treatment

- An academic center (hospital connected with a university)
- A regional center (a hospital that serves multiple cities)
- A community hospital (a hospital that is used primarily by one town or city)
- Private Practice

12. How long does it take you to reach your treating physician in person? (if you fly to your appointments, choose the answer based on how long it would take you by car).

- 0 - 1 hours
- 1 - 2 hours
- 3 - 5 hours
- 5 + hours

General Thoughts on COVID-19

Questions on this page address your thoughts on COVID-19 in general.

13. In your personal opinion, what do you believe your risk for COVID-19 is

- ◯ Lower risk than the general population
- ◯ Higher risk than the general population
- ◯ Same risk as the general population

14. Rate your anxiety about acquiring COVID-19

Not Anxious — Extremely Anxious

15. Have you contacted or tried to contact your doctor to determine if you are at higher risk for COVID-19

- ◯ Yes
- ◯ No
- ◯ I haven't been able to get a response from my doctor

16. If you have communicated with your doctor about risk; what did your doctor indicate your risk level is?

- ◯ Lower risk than the general population
- ◯ Higher risk than the general population
- ◯ Same level of risk as the general population
- ◯ My doctor didn't tell me my risk
- ◯ I haven't communicated with my doctor about risk

17. Rate your anxiety about your cancer progressing or recurring during the COVID-19 pandemic

Not Anxious — Extremely Anxious

18. What are some of the factors that you think you, or others with kidney cancer, might have that would result in higher risk?

- [ ] Having only one kidney
- [ ] Having been diagnosed with cancer
- [ ] Having metastatic disease
- [ ] Having lost part of a kidney (partial nephrectomy)
- [ ] Undergoing systemic therapy with a tyrosine kinase inhibitor (TKI, like Sutent, Votrient, lenvatinib, cabozantinib etc.)
- [ ] Being treated with immunotherapy (Opdivo, Yervoy, Keytruda)
- [ ] Being treated with an mTOR-inhibotor (Afinitor, Torisel)
- [ ] Having to visit a hospital/health-care provider
- [ ] Being treated with radiation
- [ ] Being treated with steroids for adverse events related to immunotherapy

19. What precautions are you taking?

- ○ Extreme social distancing (self-quarantine)
- ○ I am not seeing anyone outside my house except essential needs (shopping etc.)
- ○ I work in an essential field, but practicing social distancing outside of work
- ○ Seeing my family / friends outside the house but taking general precautions (remaining six feet away, coughing into sleeve, etc...)
- ○ Working at home office
- ○ No changes

20. What other precautions are you taking to protect yourself

- [ ] wearing a mask
- [ ] wearing gloves
- [ ] Other (please specify)

21. Are you willing to go to your doctor's appointment right now:

- ○ Very willing
- ○ Somewhat willing
- ○ Uncertain
- ○ Unwilling
- ○ Very unwilling

22. Please respond to each item by marking one box per row. In the past seven days...

	Never	Rarely	Sometimes	Often	Always
I felt fearful	○	○	○	○	○
I found it hard to focus on anything other than my anxiety	○	○	○	○	○
I felt uneasy	○	○	○	○	○
I felt nervous	○	○	○	○	○
I felt like I needed help for my anxiety	○	○	○	○	○
I felt anxious	○	○	○	○	○
I felt tense	○	○	○	○	○

23. Has your health care provider added additional services to reduce in-person appointments?

- [ ] Virtual appointments
- [ ] Telephone conferences
- [ ] Email exchanges
- [ ] No extra services
- [ ] Other (please specify)

About your disease

***** if you are filling out the survey on behalf of a patient please respond to all questions as if you were the patient *****

24. Rate your current level of distress

No distress ... Extreme distress

25. Please respond to each item by selecting one box in each row

	not at all	a little	somewhat	quite a bit	very much
I worry more about the possibility of cancer recurrence or progression than before	◯	◯	◯	◯	◯
I examine myself to see if I have any physical signs of cancer	◯	◯	◯	◯	◯
I examine myself to see if I have any physical signs of COVID-19	◯	◯	◯	◯	◯

* 26. Select the option that best describes your current situation

- ◯ I have been newly diagnosed with a renal mass but haven't had surgery/therapy so far
- ◯ I am in follow-up after surgery for localized disease (stage 1, 2, 3)
- ◯ I am on adjuvant therapy (therapy to prevent recurrence, but no metastatic lesions)
- ◯ I am metastatic and not on systemic therapy
- ◯ I am metastatic and on systemic therapy (drug therapy to delay or prevent progression)

Follow-Up

***** if you are filling out the survey on behalf of a patient please respond to all questions as if you were the patient *****

27. In the current environment, how willing are you to visit a healthcare facility for scans

Comments

28. When are your next scheduled scans

- ◯ Within the next 6 weeks
- ◯ Two to three months away
- ◯ In six months
- ◯ In one year

29. Are you willing to move your follow-up scans to a later date?

- ◯ Yes, delay by 1 month
- ◯ Yes, delay by 2 months
- ◯ Yes, delay by 6 months
- ◯ Delay according to my doctors recommendation
- ◯ No, I don't want to delay my scans

Newly diagnosed Renal mass

***** if you are filling out the survey on behalf of a patient please respond to all questions as if you were the patient *****

30. What size is your kidney tumor?

- ◯ smaller than 4 cm
- ◯ 4- 7 cm
- ◯ 7 - 10 cm
- ◯ more than 10 cm
- ◯ I don't know

31. Choose the option that best describes your situation

- ◯ My surgery has been postponed because of COVID-19
- ◯ My date for surgery is set and not changed
- ◯ My doctor has recommended active surveillance
- ◯ I am not sure what is going to happen, waiting for the doctor to call back
- ◯ Other (please specify)

32. About my upcoming surgery / procedure

	Strongly agree	Agree	Neither agree nor disagree	Disagree	Strongly disagree	N/A
I am anxious about moving my appointment	◯	◯	◯	◯	◯	◯
I want to move the appointment to avoid exposure to COVID-19	◯	◯	◯	◯	◯	◯
I don't want to move the appointment but I am anxious about COVID-19	◯	◯	◯	◯	◯	◯
I don't want to move the appointment and I am not worried about COVID-19	◯	◯	◯	◯	◯	◯
I am more worried about my cancer than COVID-19 and just want to focus on my cancer diagnosis	◯	◯	◯	◯	◯	◯

Systemic therapy

***** if you are filling out the survey on behalf of a patient please respond to all questions as if you were the patient *****

33. Which treatment are you currently receiving?

- ◯ Sutent (sunitinib)
- ◯ Votrient (pazopanib)
- ◯ Opdivo (nivolumab) monotherapy
- ◯ Opdivo + Yervoy (nivolumab + ipilimumab)
- ◯ Keytruda + Inlyta (pembrolizumab + axitinib)
- ◯ Bavencio + Inlyta (avelumab + axitinib)
- ◯ Lenvima + Afinitor (lenvatinib + everolimus)
- ◯ Cabometyx (cabozantinib)
- ◯ high dose Interleukin-2
- ◯ Inlyta (axitinib)
- ◯ Avastin (bevacizumab)
- ◯ Avastin + Tarceva
- ◯ Afinitor (everoliumus) monotherapy
- ◯ Nexavar (sorafenib)
- ◯ Torisel (temsirolimus)
- ◯ Other (please specify)

34. How often do you see your doctor / oncologist ?

- ◯ Every week
- ◯ Every 2 weeks
- ◯ Every 3 weeks
- ◯ Every month
- ◯ Every 3 months
- ◯ Every 6 months
- ◯ less than every 6 months

35. About my upcoming therapy:

	Strongly agree	Agree	Neither agree nor disagree	Disagree	Strongly disagree
I am anxious about moving my appointment	○	○	○	○	○
I want to move the appointment to avoid the pandemic	○	○	○	○	○
I don't want to move the appointment but I am anxious about COVID-19	○	○	○	○	○
I don't want to move the appointment and I am not worried about COVID-19	○	○	○	○	○
I am more worried about my cancer than COVID-19 and just want to focus on my cancer diagnosis	○	○	○	○	○

36. If your treatment includes an infusion, are you willing to skip an infusion?

- ○ Very willing
- ○ Willing
- ○ Uncertain
- ○ Unwilling
- ○ Very unwilling

37. Would you be willing to pause your systemic therapy?

- ○ No, I would be anxious about progression
- ○ No, I am not worried about Covid-19
- ○ Yes, for 2 months
- ○ Yes, for 3 months
- ○ Yes, as long as the doctor thinks it is safe for me
- ○ Don't know

38. If your therapy would be delayed/paused would you still want your scans?

- ○ Take place as scheduled
- ○ Also be delayed

39. Would you like your doctor to:

- [ ] Offer online counseling
- [ ] Move your appointment to a more safe date
- [ ] Communicate via email with you
- [ ] Communicate via telephone with you
- [ ] Still see you, no matter if it is urgent or not
- [ ] Don't know

Other (please specify)

40. Did your doctor change your treatment plan because of COVID-19?

- Yes - infusion delayed
- Yes - infusion paused
- Yes - TKI delayed
- Yes - TKI paused
- Yes - any treatment delayed
- Yes - any treatment paused
- No

41. Do you think your therapy is protective against COVID-19?

- Yes
- No
- Don't know

42. Do you think your therapy is increasing your risk of acquiring/having complications from COVID-19?

- Yes
- No
- Don't know

43. Rate your anxiety if you would have to pause therapy due to COVID-19?

not at all | very much so

Financial Implications

Financial toxicity describes the impact of direct and indirect health care costs that lead to significant financial burden for patients and their caregivers. These questions are aimed at better understanding financial burdens for patients.

44. Below is a list of statements that other people with your illness said are important. Please circle or mark one number per line to indicate your response as it applies to the past seven days

	Not at all	A little bit	Somewhat	Quite a bit	Very much
I know that I have enough money in savings retirements or assets to cover the cost of my treatment	○	○	○	○	○
My out-of pocket medical expenses are more than I thought they would be	○	○	○	○	○
I worry about the financial problems I will have in the future as a result of my illness or treatment	○	○	○	○	○
I feel I have no choice about the amount of money I spend on care	○	○	○	○	○
I am frustrated that I cannot work or contribute as much as I usually do	○	○	○	○	○
I am satisfied with my current financial situation	○	○	○	○	○
I am able my to meet my monthly expenses	○	○	○	○	○
I feel financially stressed	○	○	○	○	○
I am concerned about keeping my job and income, including work at home	○	○	○	○	○
My cancer or treatment has reduced my satisfaction with my present financial situation	○	○	○	○	○
I feel in control of my financial situation	○	○	○	○	○

45. I expect that the COVID-19 pandemic will worsen my financial situation

- [ ] Not at all
- [ ] A little bit
- [ ] Somewhat
- [ ] Quite a bit
- [ ] Very much

46. Do you expect the COVID-19 pandemic to induce:

	Not at all	Somehow	A lot
Medical hardship	○	○	○
Behavioral hardship	○	○	○
Psychological hardship	○	○	○
Financial hardship	○	○	○

Supplemental Information

***** if you are filling out the survey on behalf of a patient please respond to all questions as if you were the patient *****

47. What frustrates you most about COVID-19 and your cancer situation?

Thank you for your support, time and effort.

If you want to learn about kidney cancer or engage with others, please make sure to visit kccure.org for more information.
